# The Appropriateness of Testing Platelet Factor 4/Heparin Antibody in Patients Suspected of Heparin-induced Thrombocytopenia

**DOI:** 10.7759/cureus.3532

**Published:** 2018-10-31

**Authors:** Vishal Jindal, Aditi Singh, Ahmad D Siddiqui, Laszlo Leb

**Affiliations:** 1 Internal Medicine, St. Vincent Hospital, Worcester, USA; 2 Hematology and Oncology, St. Vincent Hospital, Worcester, USA

**Keywords:** 4ts scoring, platelet factor 4 (pf4), pf4 elisa test, heparin-induced thrombocytopenia (hit)

## Abstract

Heparin-induced thrombocytopenia (HIT) is an adverse reaction to the administration of heparin due to the activation of the platelets by the immunoglobulin G (IgG) antibody-platelet factor 4 (PF4)/heparin immune complex. Since the clinical outcome is uncertain (as it could be associated with significant morbidity and sometimes death), an early diagnosis and appropriate treatment are necessary. The 4Ts pretest clinical scoring system and testing for all anti-PF4/heparin antibodies can markedly improve the diagnosis and prompt adequate treatment. Our study was undertaken to retrospectively evaluate the appropriateness of ordering the PF4 enzyme-linked immunosorbent assay (ELISA) test by using the 4Ts scoring system in a tertiary institution. We examined a database of 118 patients who had the PF4 ELISA test and calculated their 4Ts scores retrospectively. A total of 107 patients were evaluated; 95 patients (88.79%) had a negative PF4 ELISA assay and 12 patients tested positive (11.21%). Only one patient tested weakly positive in the low probability group (negative predictive value 98%). In the intermediate group, six patients were strongly positive (optical density (OD) > 1.0). In this latter group, further confirmatory testing using serotonin release assays (SRAs) could have been done. We also evaluated the setting where the tests were performed and found that the majority of patients (63.55%) were tested in the intensive care unit (ICU) where thrombocytopenia is multifactorial. We concluded that the large majority of patients were not appropriately evaluated prior to testing, which incurred unnecessary expense and patient distress. For the proper identification of patients suspected of HIT who should undergo PF4/heparin antibody testing, further education of the ordering physicians is recommended.

## Introduction

Heparin-induced thrombocytopenia (HIT) is an important and serious complication of heparin treatment. The first case of arterial thrombosis associated with heparin treatment was described in the 1950’s [1]; however, thrombocytopenia as a common occurrence in patients treated with heparin was recognized only in 1970 [2]. In the late 1970’s, the clinical features of HIT were defined [3]. The demonstration that HIT is caused by platelet factor 4 (PF4)/heparin antigen-antibody complexes [4-5] led to the development of a PF4 enzyme-linked immunosorbent assay (PF4-ELISA) and to the more specific serotonin release functional assay. The evaluation of the clinical probability of HIT was significantly improved by introducing in practice the 4Ts scoring system, which has a high negative predictive value [6].

The easy availability of an automated platelet count prompted physicians to suspect excessive HIT in heparinized patients who developed thrombocytopenia. Furthermore, the PF4-ELISA assay, being relatively easily obtainable, was used to confirm the diagnosis. In the last decade, the almost universally applied 4Ts scoring system and the demonstration of the low specificity of PF4-ELISA assay were expected to further improve the diagnosis of clinically suspected HIT. The present study was undertaken to retrospectively evaluate the appropriateness of ordering the PF4 ELISA test in clinically suspected HIT by using the 4Ts scoring system as a pretest tool in a tertiary hospital. We hope that this study will further contribute to a more judicious diagnosis and management of patients suspected of HIT.

## Materials and methods

In this study, we did a retrospective chart review of patients at Saint Vincent Hospital, Worcester who had a PF4 ELISA assay done for HIT during the in-hospital stay. After the Metrowest Medical Center Institutional Review Board approval, a total of 118 hospitalized patients from January 1, 2015 to June 30, 2017 were enrolled in this study. Out of that, 11 patients were removed - eight due to the lack of clinical data and three due to doubling, as they had a repeat test within three months. A chart review, including laboratory data and radiological data, was used to calculate the fall in the platelet count, exposure to heparin, any thrombotic event, and the presence of other causes of thrombocytopenia. From this data, we calculated the 4Ts score and divided patients into three groups: low probability - 4Ts score 0 to 3, intermediate probability - 4Ts score 4 - 5, and high probability - 4Ts score ≥ 6. The PF4 ELISA antibody results with optical density (OD) values were divided into three groups, 0 to 0.4 (negative), 0.4 to 1 (weakly positive), and ≥ 1 (strongly positive). Along with that, additional data was integrated regarding the demographics, diagnosis, and site of admission (Table 1). The PF4 ELISA assay for heparin antibodies was performed according to the manufacturer’s specifications.

**Table 1:** Demographics, Diagnosis, and Setting CABG: coronary artery bypass grafting; ICU: intensive care unit; MI: myocardial infarction

	Mean
Age	71.25 ± 11.81
Gender	Frequency (percentage)
Male	52 (48.60%)
Female	55 (51.40%)
Major Diagnosis	
Sepsis	40 (37.38%)
Malignancy	14 (13.08%)
MI/CABG/cardiogenic shock	15 (14.02%)
Intestinal perforation	10 (9.35%)
Renal failure	21 (19.63%)
Pneumonia and other respiratory issues	26 (24.30%)
Others	10 (9.35%)
Settings	
ICU	68 (63.55%)
Non-ICU	39 (36.45%)

## Results

Demographic data

A total of 118 patient’s profiles were analyzed retrospectively, and out of that number, 11 were excluded from the study. Eight patients were excluded due to the lack of data and three due to doubling as they had a repeat test within three months. Thus, our total sample size was 107. In the study population, the mean age was 71.25 ± 11.81, with a minimum age of 43 and maximum age of 96 (95% confidence interval (CI) 68.99 to 73.52) (Table 1). The total of male participants was 52 (48.60%) and the remaining 55 (73.47%) were female participants. Among the study population, 51 (47.66%) were low probability, 51 (47.66%) were intermediate probability, and five (4.67%) were high probability.

Intensive care unit (ICU) admission and diagnosis

Sixty-eight out of 107 patients were in the ICU, and in further subgroup division, 30 (44%) belonged to the low probability group, 36 (53%) belonged to the intermediate probability group, and two (3%) belonged to high probability group. The difference in the proportion of total 4Ts score group between the three subgroups in ICU was not statistically significant (P value - 0.249). Among the study population, 40 (37.38%) had sepsis. The number of people with malignancy, MI/CABG/cardiogenic shock, intestinal perforation, renal failure, pneumonia and other respiratory issues, and other diagnosis was 14 (13.08%), 15 (14.02%), 10 (9.35%), 21 (19.63%), 26 (24.30%), and 10 (9.35%), respectively.

PF4 ELISA HIT antibody and 4Ts scoring

**Figure 1:**
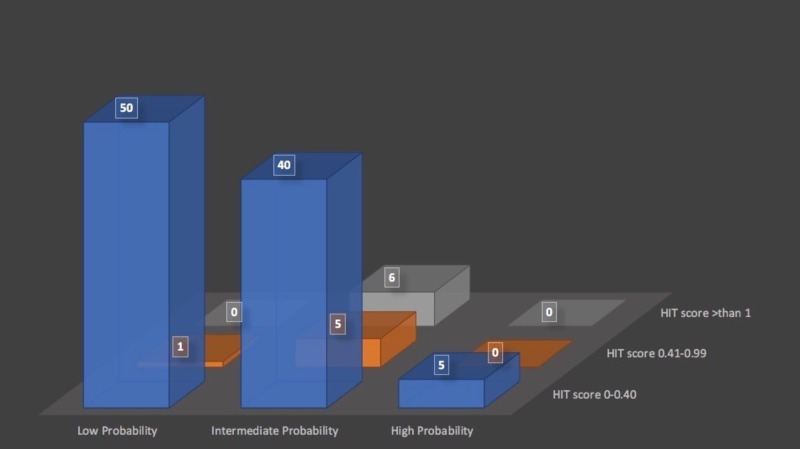
Comparison of 4Ts Score and PF4 ELISA Results HIT: heparin-induced thrombocytopenia; PF4 ELISA: platelet factor 4 enzyme-linked immunosorbent assay

Out of the total sample size, 95 patients (88.79%) were negative for the HIT antibody and only 12 patients (11.21%) were positive (OD > 0.40). The patients with the positive PF4 HIT ELISA antibody were further classified into two groups on the basis of the OD value: weakly positive (OD value: 0.40 - 0.99) and strongly positive (OD: > 1.00). There were six patients (5.61%) in each group, as shown in Table 2. On comparing the 4Ts score with OD values, we found only one patient tested weakly positive in low probability in the 4Ts score group and the rest were negative, which gives a negative predictive value of the 4Ts scoring system in the low probability group of 98% (Figure 1). The other 11 patients were in the intermediate probability group, and there was no patient with a PF4-HIT antibody in the high probability group. There were only six patients who had an OD value of more than 1. Therefore, the positive predictive value of this test was 19.6%.

**Table 2:** 4Ts Scoring and PF4 ELISA Results OD: optical density; PF4-ELISA: platelet factor 4 enzyme-linked immunosorbent assay

Total 4Ts score group	
Low probability (0 - 3)	51 (47.66%)
Intermediate probability (4 - 5)	51 (47.66%)
High probability (≥ 6)	5 (4.67%)
PF4 ELISA OD results	
0 - 0.40 (Negative)	95 (88.79%)
0.41 - 0.99 (weakly positive)	6 (5.61%)
> 1.0 (Strongly positive)	6 (5.61%)

## Discussion

The diagnosis of HIT is achieved by taking into account both clinical and laboratory data. The clinical pre-test evaluation was improved by the use of the 4Ts clinical scoring system which divides patients into the low probability 4Ts score (0 - 3), intermediate probability (4 - 5), and high probability (≥ 6). Low 4Ts scores were associated with a high negative predictive value (0.998) [7]. The intermediate and high scores had a poor positive predictive value for HIT (0.14 and 0.64, respectively) [7]. Based on these findings, the American Society of Haematology recommends against testing for HIT in patients with low pre-test probability but testing when the pre-test probability is > 8%. This will include patients with intermediate or high 4Ts score. The laboratory testing for HIT is performed in most hospitals using the polyspecific PF4 ELISA assay, which is widely available, offers a rapid turnaround time, and is highly sensitive (> 99%) but has a low specificity (30% to 70%) [8]. Using the quantification of the test with the OD value, it has been reported that the specificity improved significantly at the value of OD > 1.0 [9]. This strongly positive higher value correlates significantly with the “gold standard” serotonin release assays (SRAs).

In this retrospective study, only 12 patients (11.21%) out of 107 tested positive in the polyspecific PF4 ELISA assay having an OD value of > 0.40, which is in the range of previously published studies at 3% and 17% [10-11]. However, only six patients (5.6%) had a PF4 ELISA OD value > 1.0 which could correlate well the SRA and thus could be considered true positive. This value is still in the range of previous studies. In the low probability category of clinical suspicion, the negative predictive value was 98%, which is similar to data published in the literature [11], and this is the group in which no testing should have been performed. Only six patients in the intermediate probability category of clinical suspicion for HIT tested positive with an OD value > 1.0, which shows the positive predictive value of the 4Ts scoring system is low in patients of the intermediate probability group. Since the positive test in this group increases the post-test probability of HIT to 40% to 64% [7], an SRA could have been indicated to confirm HIT or if the clinical condition necessitated administering direct thrombin inhibitors (DTI). Regarding the five patients in the high probability category of clinical suspicion in the 4Ts score, the positive predictive value is 0.64 and the negative PF4 ELISA assay was most likely due to the low number of tested patients. In case the suspicion for HIT remains high in such patients, they should probably be placed on DTI.

Regarding the setting of evaluated patients, the large majority of patients were from the ICU (63.45%). This is of no surprise as in this setting many patients receive heparin treatment and they also often had thrombocytopenia. However, identification of the cause of thrombocytopenia in ICU is difficult as patients can develop thrombocytopenia as a result of multiple aetiologies, including liver disease, drugs, sepsis, or disseminated intravascular coagulation, making it difficult to attribute the low platelet count to heparin administration only. The fact that the number of beds in the ICU is relatively few and the patient turnover is low compared to the medical and surgical settings further emphasizes the disproportionate number of PF4 ELISA tests ordered in the ICU. The majority of patients were diagnosed with sepsis (38%) and respiratory illnesses (24.30%). All patients were recruited from the ICU, which further supports the difficulty to attribute the cause of thrombocytopenia to heparin treatment only.

## Conclusions

Thus, this study confirms previous ones about the importance of implementing the 4Ts scoring system in clinical practice and limiting testing for PF4/heparin antibody assays only to patients with intermediate and high 4Ts values. By implementing such clinical guidelines, the number of ordered tests can be cut almost in half (which is also of economic value). It is of interest that none of the tested patients, particularly those with intermediate and high 4Ts, had their heparin therapy discontinued or changed to DTI. This was probably due to either non-reliance on the clinical evaluation or disregarding the test results. Our study demonstrates an insufficient education of the medical staff regarding the importance of pre-test clinical evaluation and the correct ordering of tests for PF4-heparin antibodies testing. Education of physicians regarding diagnosing and treating HIT is badly needed, particularly in the settings of ICU where the majority of inappropriate tests were ordered. 
